# Supplementary material for: The causal relationship between the human gut microbiota and pyogenic arthritis: a Mendelian randomization study
Source: Front Cell Infect Microbiol. 2024 Nov 26;14:1452480. doi: 10.3389/fcimb.2024.1452480 (PMC11629706; doi:10.3389/fcimb.2024.1452480)
Supplement: Supplementary file 1 [file DataSheet1.zip › mendelian test/ebi-a-GCST90027627.csv_leave_one_out.pdf]

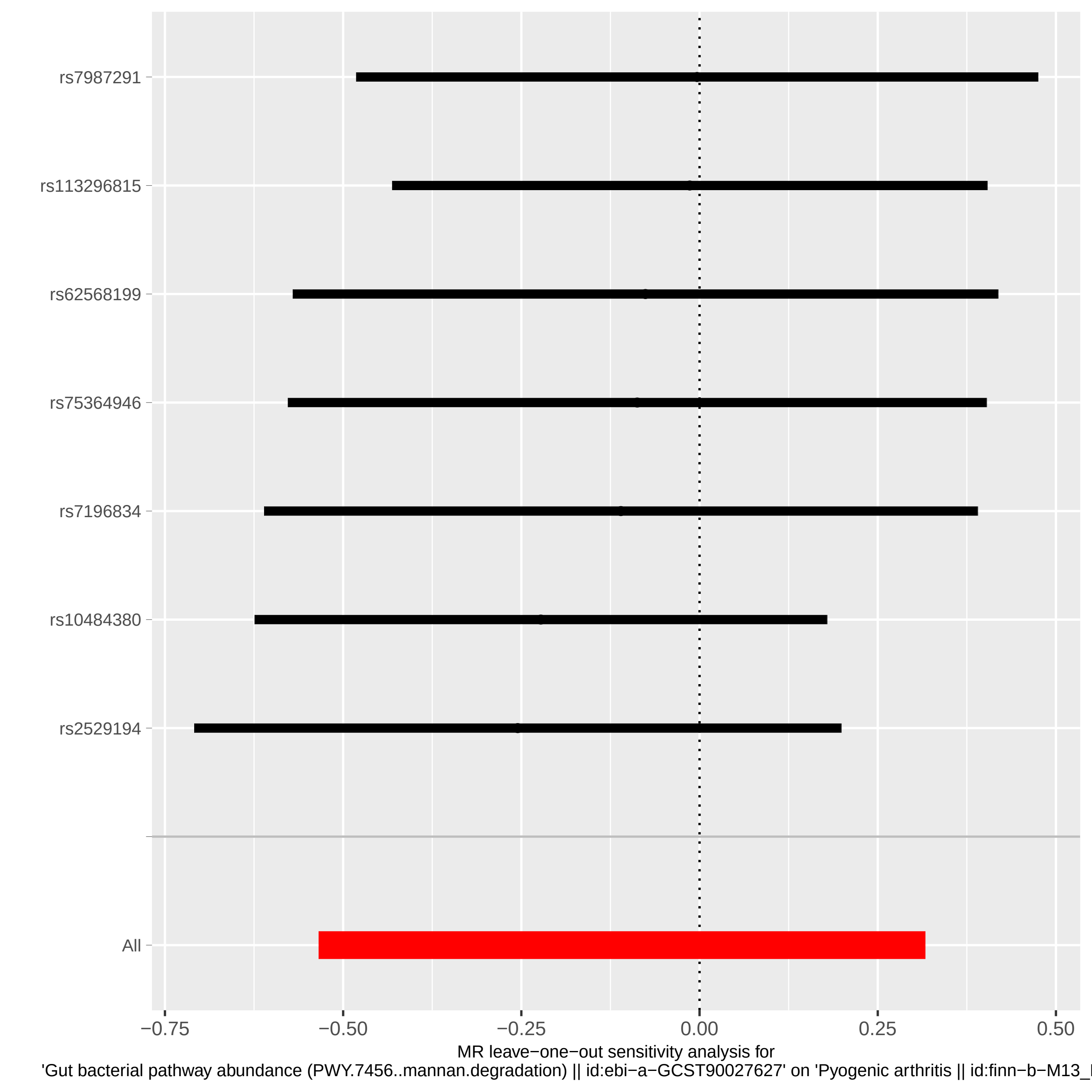
rs7987291
rs113296815
rs62568199
rs75364946
rs7196834
rs10484380
rs2529194
All
−0.75
−0.50
−0.25
0.00
0.25
0.50
MR leave−one−out sensitivity analysis for
'Gut bacterial pathway abundance (PWY.7456..mannan.degradation) || id:ebi−a−GCST90027627' on 'Pyogenic arthritis || id:finn−b−M13_
